# Supplementary material for: Deciphering Cell Cycle Dynamics and Cell States in Single-cell RNA-seq data with SPAE
Source: bioRxiv. 2026 Mar 8:2026.03.05.709782. Preprint. [Version 1] doi: 10.64898/2026.03.05.709782 (PMC12991139; doi:10.64898/2026.03.05.709782)
Supplement: Supplement 1 — Supplementary Figure S1. Performance of CYCLOPS on pseudotime inference. (A) Scatter plots showing the Pearson correlation between gene expression and pseudotime for the top six genes identified by CYCLOPS. (B) Pearson correlation between gene expression and pseudotime for known cell cycle markers Aurka, Cdca2, and Kpna2 as inferred by CYCLOPS. Supplementary Figure S2. Performance of Cyclum on pseudotime inference. (A) Scatter plots showing the Pearson correlation between gene expression and pseudotime for the top six genes identified by Cyclum. (B) Pearson correlation between gene expression and pseudotime for known cell cycle markers Aurka, Cdca2, and Kpna2 as inferred by Cyclum. Supplementary Figure S3. Performance of reCAT on pseudotime inference. (A) Scatter plots showing the Pearson correlation between gene expression and pseudotime for the top six genes identified by reCAT. (B) Pearson correlation between gene expression and pseudotime for known cell cycle markers Aurka, Cdca2, and Kpna2 as inferred by reCAT. Supplementary Figure S4. Performance of Monocle on pseudotime inference. (A) Scatter plots showing the Pearson correlation between gene expression and pseudotime for the top six genes identified by Monocle. (B) Pearson correlation between gene expression and pseudotime for known cell cycle markers Aurka, Cdca2, and Kpna2 as inferred by Monocle. Supplementary Figure S5. Benchmarking classification metrics on mESCs Quartz-Seq data. Radar chart displaying seven multi-class classification metrics (Fscore, Recall, Precision, Accuracy, NMI, ARI, RI) evaluated on the mESCs Quartz-Seq dataset. The performance of SPAE is compared against Cyclum, CYCLOPS, Seurat, Cyclone, and reCAT. Supplementary Figure S6. Robustness analysis under gene subsampling. Boxplots of Fscore, Precision, Recall, and RI metrics indicating the performance of SPAE, CYCLOPS, and Cyclum on subsampled datasets with varying numbers of genes (ranging from 50 to 600). Supplementary Figure S7. Rob [file media-1.pdf]

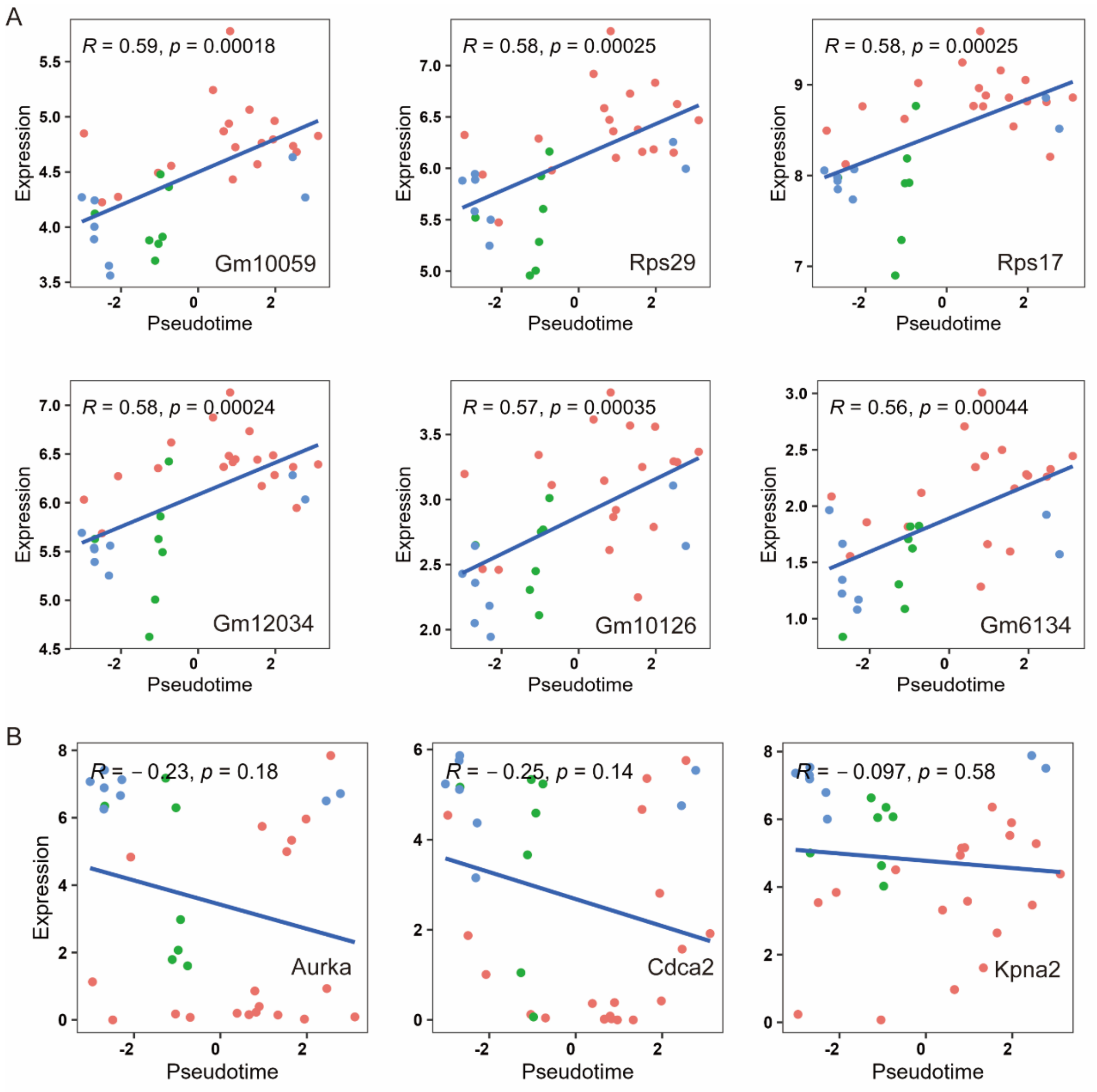

Supplementary Figure S1. **(A)** Pearson correlation between gene expression and pseudotime for the top six genes identified by CYCLOPS. **(B)** Pearson correlation between gene expression and pseudotime for *Aurka*, *Cdca2*, and *Kpna2* by CYCLOPS.

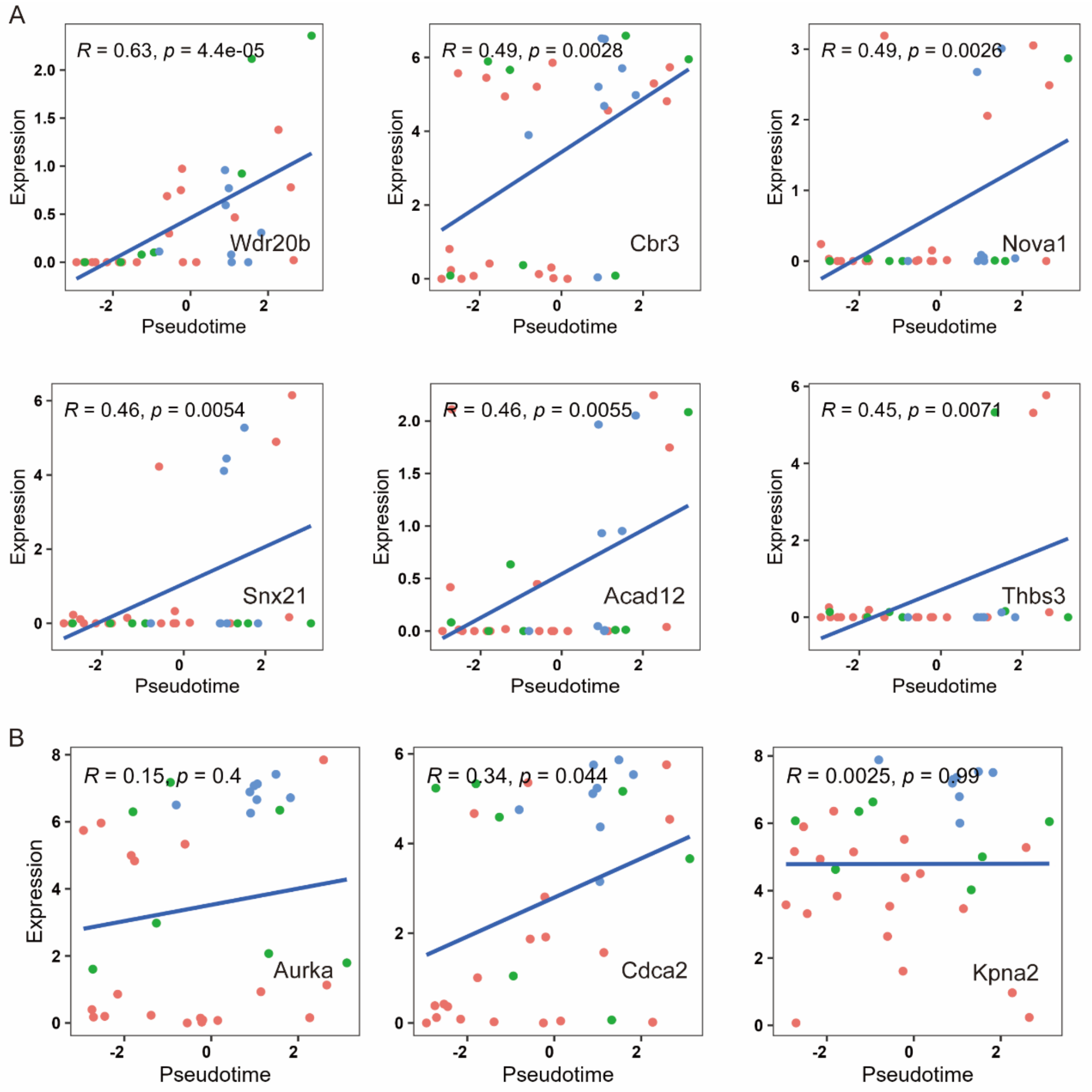

Supplementary Figure S2. **(A)** Pearson correlation between gene expression and pseudotime for the top six genes identified by Cyclum. **(B)** Pearson correlation between gene expression and pseudotime for *Aurka*, *Cdca2*, and *Kpna2* by Cyclum.

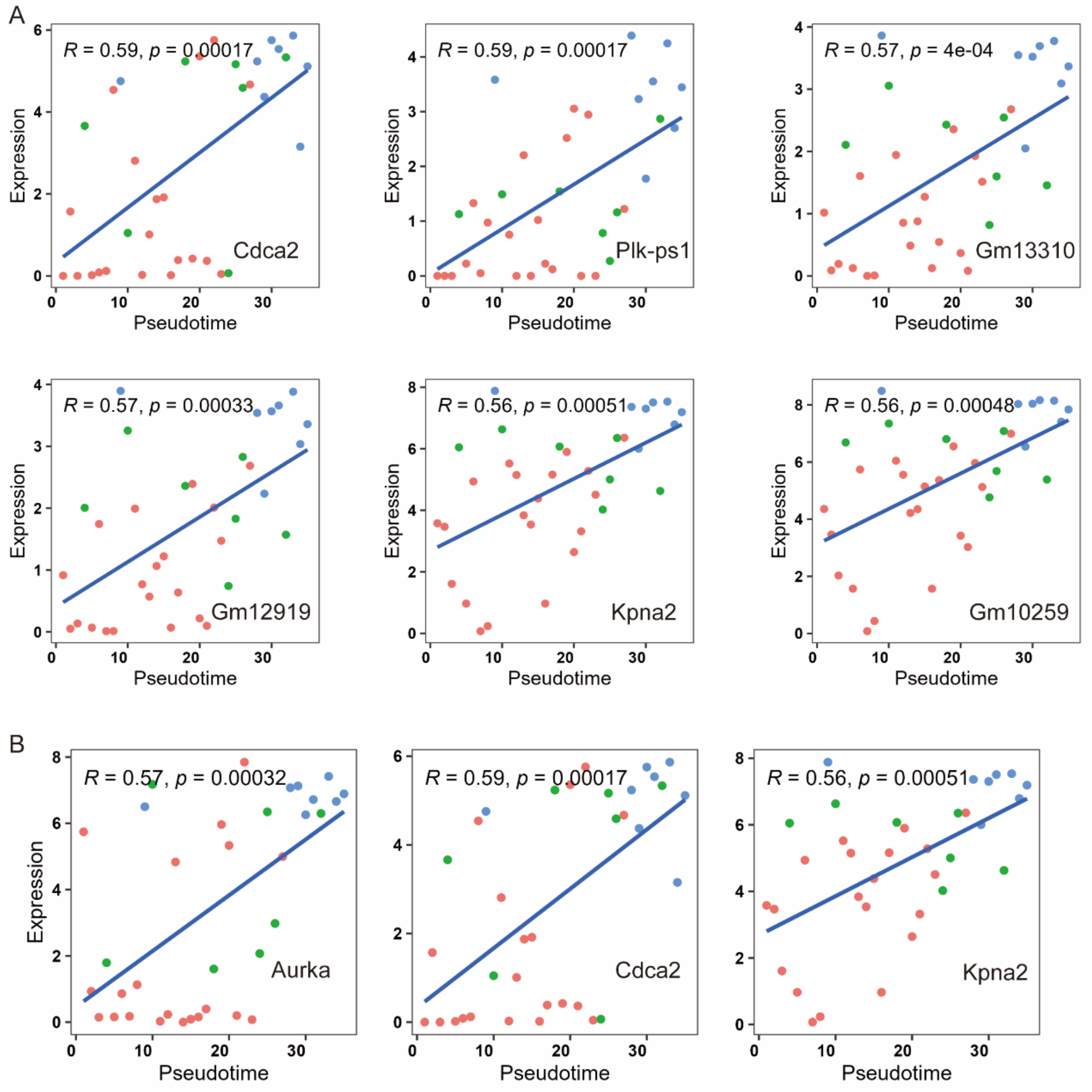

Supplementary Figure S3. **(A)** Pearson correlation between gene expression and pseudotime for the top six genes identified by reCAT. **(B)** Pearson correlation between gene expression and pseudotime for *Aurka*, *Cdca2*, and *Kpna2* by reCAT.

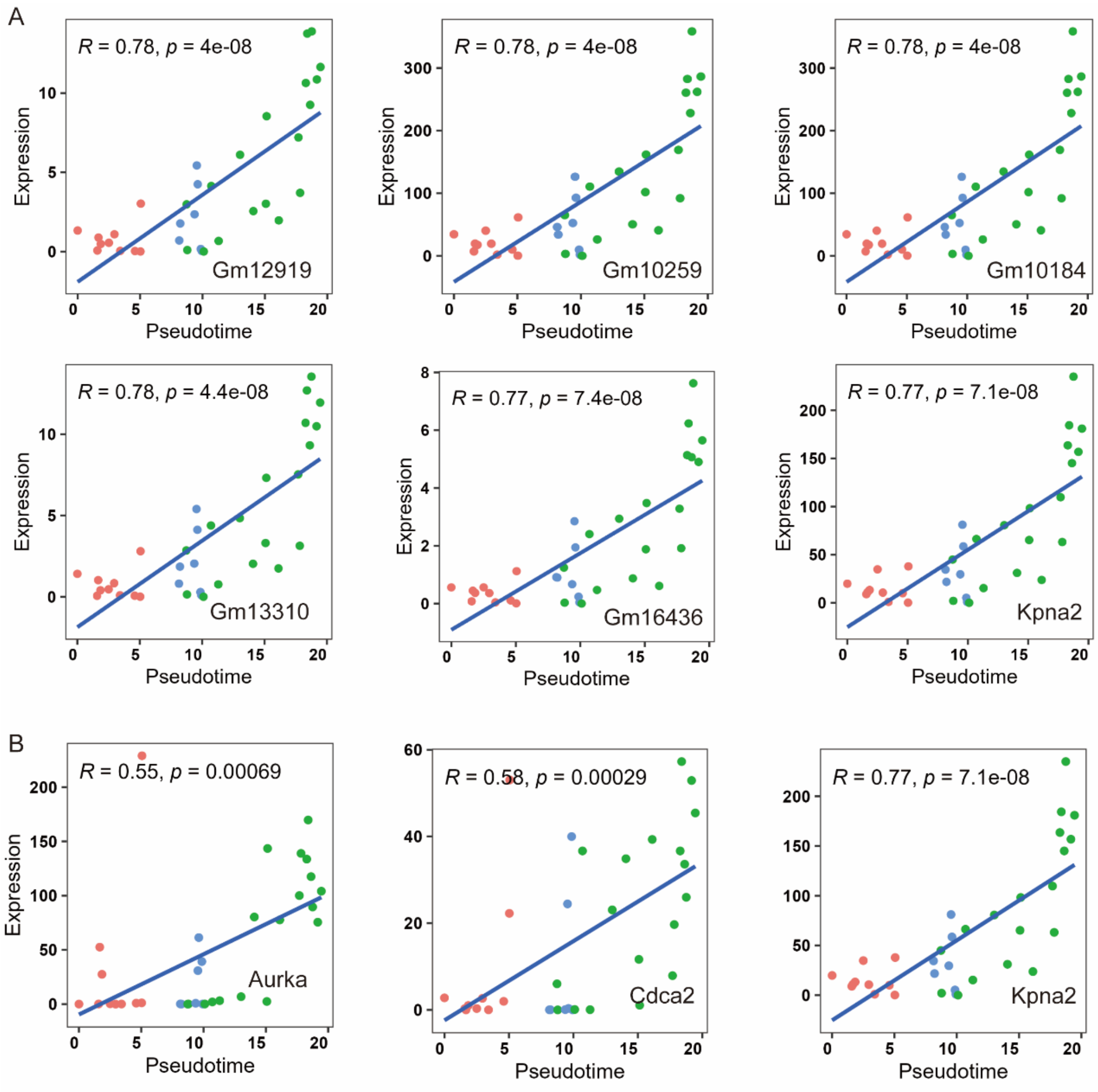

Supplementary Figure S4. **(A)** Pearson correlation between gene expression and pseudotime for the top six genes identified by Monocle. **(B)** Pearson correlation between gene expression and pseudotime for *Aurka*, *Cdca2*, and *Kpna2* by Monocle.

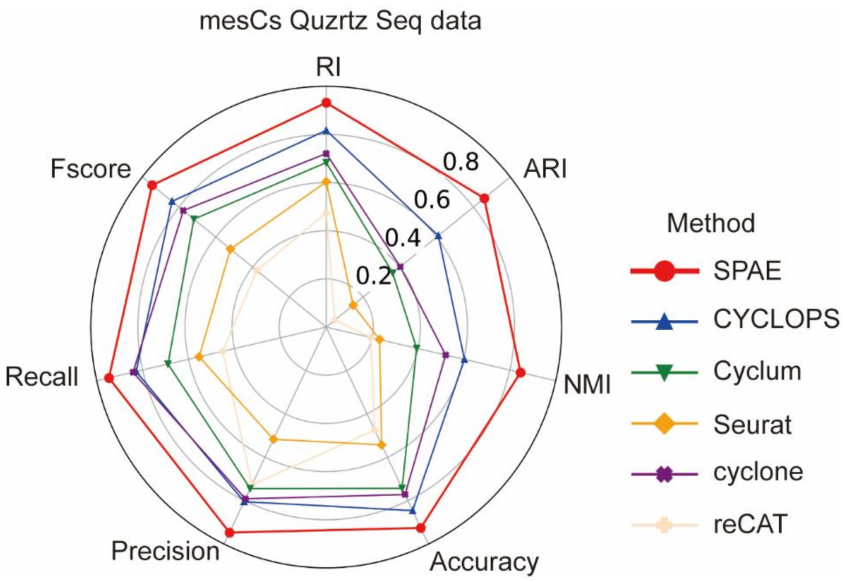

Supplementary Figure S5. Radar chart shows seven multi-class classification metrics used to evaluate the cell cycle classification accuracy of SPAE, cyclone, Seurat, reCAT, Cyclum, and CYCLOPS on mesCs Quartz Seq data.

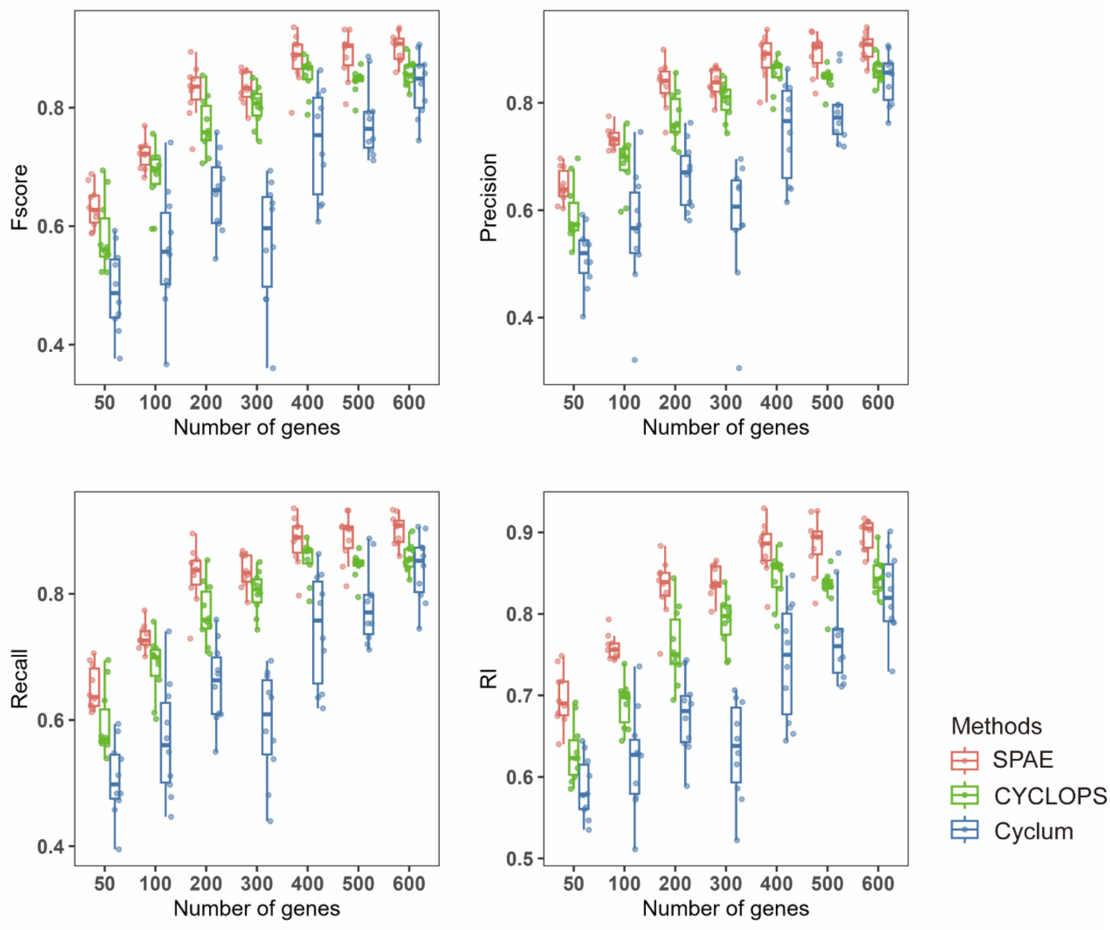

Supplementary Figure S6. Boxplots of Fscore, Precision, Recall and RI values indicate the performance of SPAE CYCLOPS and Cyclum on the subsampled datasets with different number of genes.

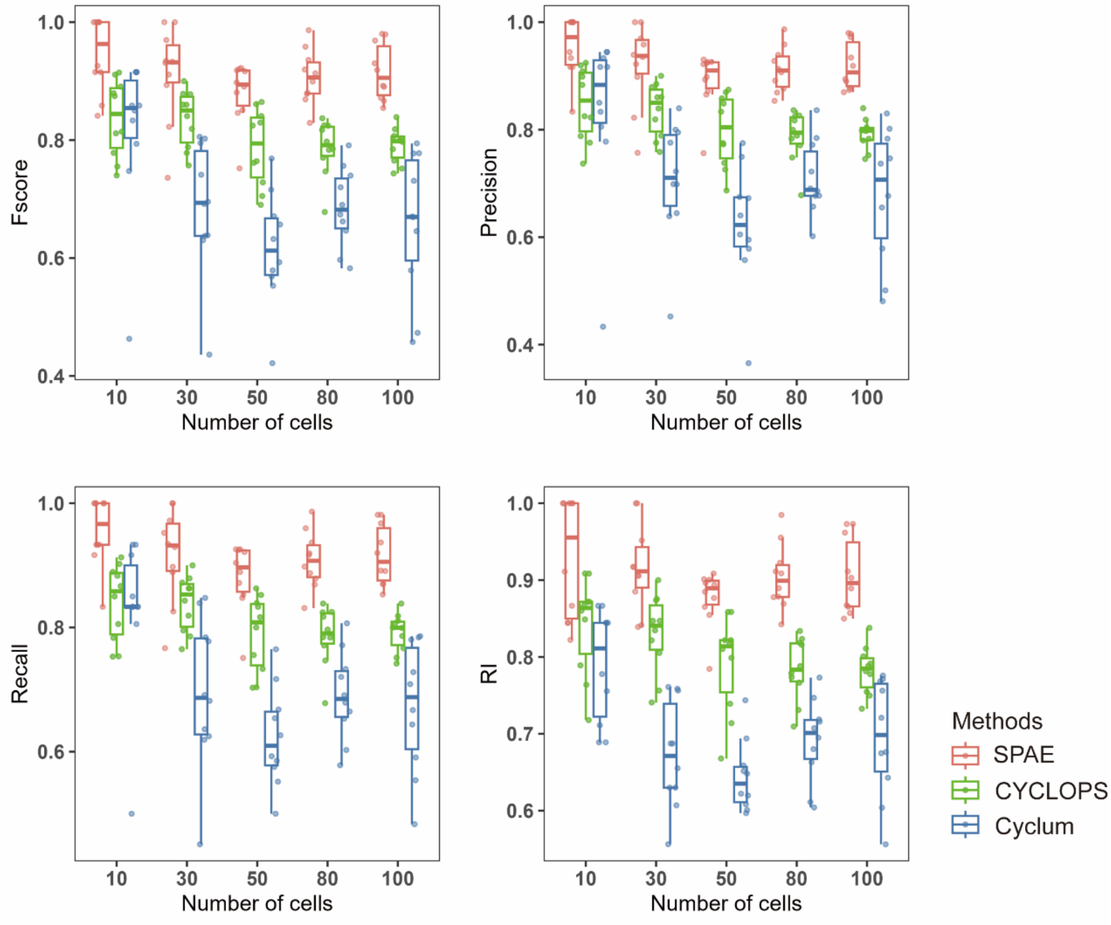

Supplementary Figure S7. Boxplots of Fscore, Precision, Recall and RI values indicate the performance of SPAE CYCLOPS and Cyclum on the subsampled datasets with different numbers of cells.

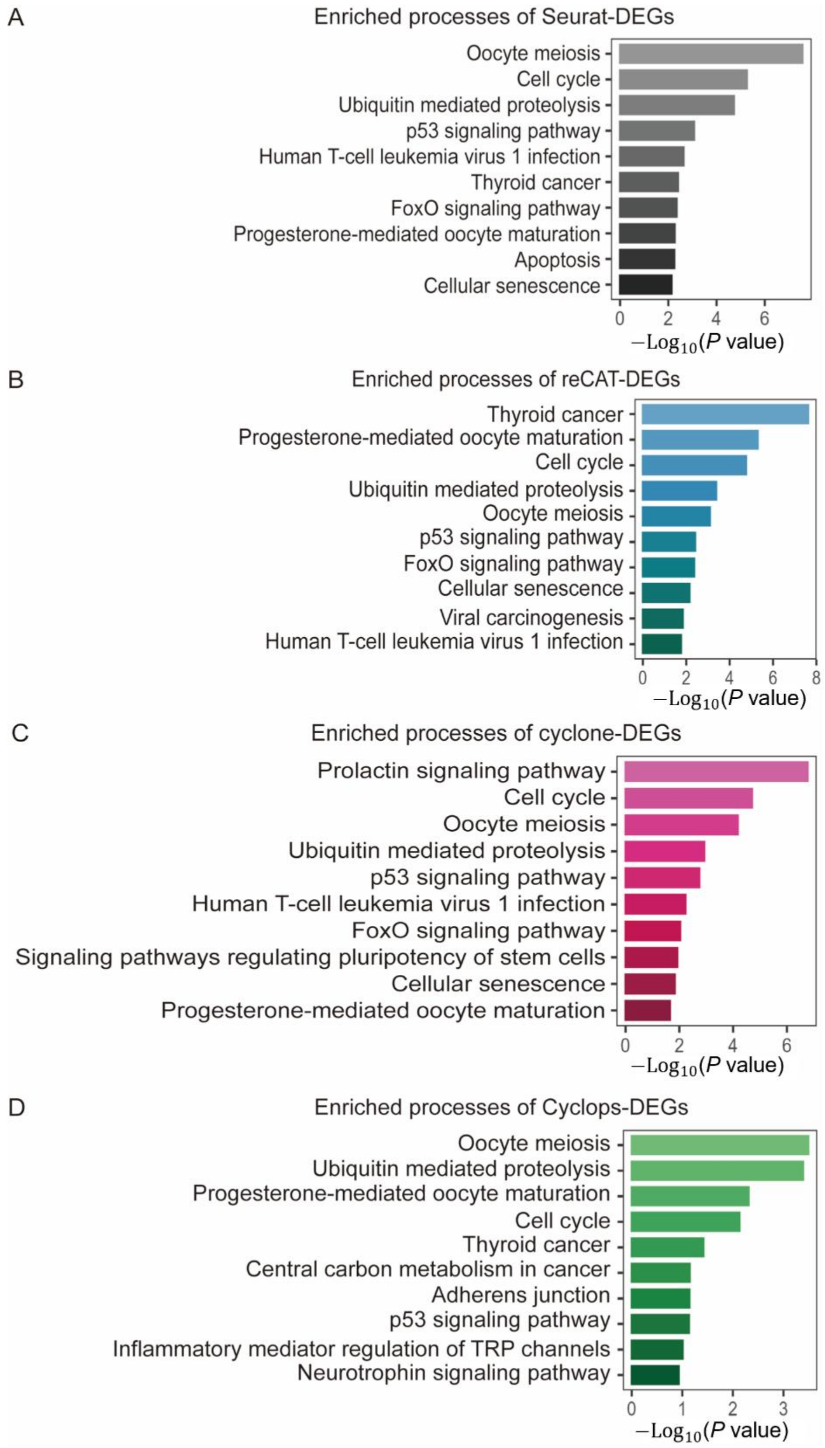

Supplementary Figure S8. Top ten enriched biological processes associated with DEGs identified by cell cycle stages inferred through Seurat, reCAT, cyclone and CYCLOPS.

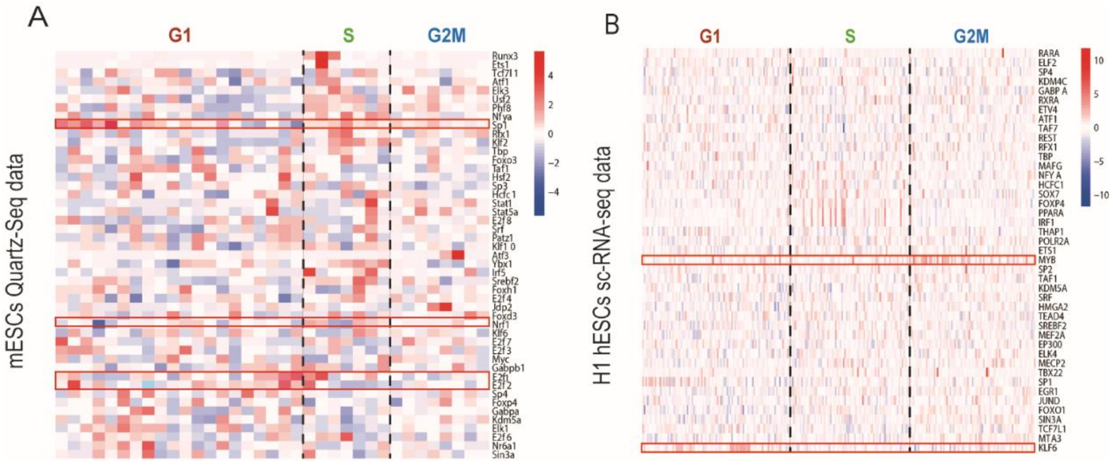

Supplementary Figure S9. SPAE predictions of core transcription factor activity during the cell cycle. **(A)** Motif activity of transcription factors in the cell cycle in the mESCs Quartz-Seq dataset. **(B)** Motif activity of transcription factors in the cell cycle in the hESCs scRNA-seq dataset. The color changes in the heatmap represent the variation in transcription factor activity, ranging from low (blue) to high (red).
