# Supplementary material for: Deciphering Cell Cycle Dynamics and Cell States in Single-cell RNA-seq data with SPAE
Source: bioRxiv. 2026 Mar 8:2026.03.05.709782. Preprint. [Version 1] doi: 10.64898/2026.03.05.709782 (PMC12991139; doi:10.64898/2026.03.05.709782)
Supplement: Supplement 2 — Supplementary Table S1. Summary of computational methods for single-cell cell cycle analysis compared in this study. [file media-2.pdf]

Supplementary Table S1. Summary of computational methods for single-cell cell cycle analysis compared in this study.

Methods	Architecture	Focus	functions
CCPE	3D helical embedding with linear and nonlinear optimization	Captures continuous cyclic trajectories (linear assumption)	Reconstructs cell cycle progression from scRNA-seq data
cyclone	Pairwise gene ranking + SVM classification	Captures periodic structure based on known marker pairs	Assigns cell cycle phases (G1, S, G2M)
Seurat	Marker gene scoring (S.Score, G2M.Score)	Infers rhythmic transcriptomic variation via gene sets	Scores and classifies cell cycle phases
reCAT	Spectral clustering + Traveling Salesman Problem (TSP)	Recovers full cyclic ordering from unsynchronized data	Reconstructs temporal order of cycling cells
Cyclum	Autoencoder with sinusoidal activation (sin − cos latent space)	Infers nonlinear cyclic trajectories (Unsupervised)	Learns cyclic gene expression patterns in an unsupervised manner
CYCLOPS	Cyclic optimization with periodic basis decomposition	Orders cells along a closed elliptical curve	Estimates pseudotime along a cyclic trajectory
ccRemover	Principal component removal (unsupervised)	Focuses on eliminating cell cycle confounding effects	Removes cell cycle − associated variation from expression matrix

Note: SVM, Support Vector Machine; TSP, Traveling Salesman Problem; scRNA-seq, single-cell RNA sequencing.
